# Design of a medical decision-supporting system for the identification of brain tumors using entropy-based thresholding and non-local texture features

**DOI:** 10.3389/fnhum.2023.1157155

**Published:** 2023-03-22

**Authors:** K. Rasool Reddy, Raj Kumar Batchu, Srinivasu Polinati, Durga Prasad Bavirisetti

**Affiliations:** ^1^Department of Electronics and Communication Engineering, Malla Reddy College of Engineering and Technology (MRCET), Hyderabad, India; ^2^Department of Computer Science and Engineering (Data Science), Prasad V. Potluri Siddhartha Institute of Technology (PVPSIT), Vijayawada, India; ^3^Department of Electronics and Communication Engineering, Vignan’s Institute of Engineering for Women (VIEW), Visakhapatnam, India; ^4^Department of Computer Science, Norwegian University of Science and Technology (NTNU), Trondheim, Norway

**Keywords:** brain tumors, entropy, magnetic resonance imaging, non-local binary pattern, thresholding, tuned single-scale retinex

## Abstract

**Introduction:**

Brain tumors arise due to abnormal growth of cells at any brain location with uneven boundaries and shapes. Usually, they proliferate rapidly, and their size increases by approximately 1.4% a day, resulting in invisible illness and psychological and behavioral changes in the human body. It is one of the leading causes of the increase in the mortality rate of adults worldwide. Therefore, early prediction of brain tumors is crucial in saving a patient’s life. In addition, selecting a suitable imaging sequence also plays a significant role in treating brain tumors. Among available techniques, the magnetic resonance (MR) imaging modality is widely used due to its noninvasive nature and ability to represent the inherent details of brain tissue. Several computer-assisted diagnosis (CAD) approaches have recently been developed based on these observations. However, there is scope for improvement due to tumor characteristics and image noise variations. Hence, it is essential to establish a new paradigm.

**Methods:**

This paper attempts to develop a new medical decision-support system for detecting and differentiating brain tumors from MR images. In the implemented approach, initially, we improve the contrast and brightness using the tuned single-scale retinex (TSSR) approach. Then, we extract the infected tumor region(s) using maximum entropy-based thresholding and morphological operations. Furthermore, we obtain the relevant texture features based on the non-local binary pattern (NLBP) feature descriptor. Finally, the extracted features are subjected to a support vector machine (SVM), K-nearest neighbors (KNN), random forest (RF), and GentleBoost (GB).

**Results:**

The presented CAD model achieved 99.75% classification accuracy with 5-fold cross-validation and a 91.88% dice similarity score, which is higher than the existing models.

**Discussions:**

By analyzing the experimental outcomes, we conclude that our method can be used as a supportive clinical tool for physicians during the diagnosis of brain tumors.

## 1. Introduction

Magnetic resonance (MR) imaging is a widely used noninvasive imaging sequence for the visualization of various brain abnormalities since it offers high-contrast human brain tissue images (Westbrook, 2014). Hence, researchers are working extensively on MR images to identify brain abnormalities and other soft tissue details in medical applications (Ullah et al., 2020). There are many methods have been proposed in recent years for detecting tumors from brain MR images. Among them, computer-aided diagnosis (CAD) based approaches have received significant attention since they speed up the diagnostic process and substantially minimize manual intervention (Tiwari et al., 2020). Typically, these techniques include pre-processing, feature extraction, feature selection and reduction, and classification (Abd-Ellah et al., 2016).

Pre-processing is a fundamental step in the analysis of brain MR images. In this phase, noise and other artifacts are removed from the MR brain images to make them suitable for subsequent stages. It can be achieved by using image enhancement approaches (Garg and Juneja, 2019). Usually, these techniques improve the image’s visual quality, and one can easily obtain information.

In the feature extraction phase, identifying the significant features present in the brain MR images results in better knowledge about the image. Usually, these features capture the inherent details of the source images. Texture feature extraction techniques are popular among the available methods, including statistical and structural, transform, model, graph, learning, and entropy-based methods (Humeau-Heurtier, 2019).

Feature selection and reduction techniques play a vital role in improving the accuracy of classification. Typically, they reduce the number of features or attributes in a dataset. These two approaches are used for the same purpose, but they have significant differences. Feature selection excludes features with missing values, low variance attributes, and highly correlated attributes without changing them. At the same time, feature reduction simplifies the problem space from higher to lower dimensions. The most frequently used reduction methods are: principal component analysis (PCA), kernel PCA (KPCA), probabilistic PCA (PPCA), linear discriminant analysis (LDA), etc. However, these are optional for models where the features are limited.

Finally, the selected or reduced attributes are applied to a classifier at the classification level to distinguish between healthy and pathological brain MR images. Recently, in most of the literature, supervised learning approaches such as support vector machine (SVM), K-nearest neighbors (KNN), logistic regression (LR), naive Bayes (NB), boosting and bagging, random forest (RF), and neural network-based classifiers have been used for adequate classification.

Based on the above discussion, recently, several CAD methods have been introduced (Mohan and Subashini, 2018; Rao and Karunakara, 2021). We discuss a few of the latest strategies and summarize the techniques’ limitations.

Mudda et al. (2020) implemented an improved brain tumor classification framework based on the gray-level run-length matrix (GLRLM), center-symmetric local binary patterns (CS-LBP), and artificial neural network (ANN). Sumathi and Mandadi (2021) developed an automated segmentation approach using kernel-based probabilistic C-means (KPCM), particle swarm optimization (PSO) and morphological operations. Paul and Sivarani (2020) designed a CAD approach to detect MR-based brain tumors using fuzzy K-means clustering (FKM), gray-level co-occurrence matrix (GLCM), and a bag of visual word (BOVW) classifier. Lu et al. (2018) proposed a novel framework for the early detection of brain tumors using wavelet energy features and a kernel-based extreme learning machine (K-LEM).

Rajesh et al. (2019) suggested a novel system for the efficient segmentation and classification of brain MR images using rough set theory (RST) and a particle swarm optimization-based neural network (PSONN). Veeramuthu et al. (2022) developed a combined feature and image-based classifier (CFIC) for the classification of brain MR images. Kanmani and Marikkannu (2018) developed a CAD system for improving classification and segmentation accuracy using threshold-based region optimization (TBRO) and corner detection approaches. Selvapandian and Manivannan (2018) proposed an automated brain tumor segmentation approach using GLCM, GLRLM, and gradient boosting. Habib et al. (2021) implemented a hybrid methodology for detecting and classifying MR-based brain tumors.

Sumathi et al. (2021) suggested an automated methodology for the early detection of brain tumors based on cuckoo search and the KNN classifier. Singh et al. (2020) designed a hybrid framework using the discrete wavelet transform (DWT), independent component analysis (ICA), and SVM. Sandhya et al. (2020) proposed an efficient brain tumor segmentation approach using a self-organizing map (SOM)-based active contour model (SOM-ACM). Polepaka et al. (2020) developed an enhanced CAD approach using local-binary patterns (LBP) and an SVM classifier.

Hua et al. (2021) presented a novel brain tumor segmentation approach using an improved multiview fuzzy C-means clustering algorithm. Younis et al. (2022) implemented an ensemble learning model for the early diagnosis of MR-based brain tumors. Mandle et al. (2022) suggested an automated detection framework for segmenting and classifying brain tumors in MR images using DWT, PCA, and kernel-based SVM (K-SVM). Behera et al. (2022) developed an ensemble deep transfer learning model using simple linear iterative clustering (SLIC) and convolutional neural networks (CNN).

Rupesh kumar et al. (Kolla et al., 2022) suggested an intelligent design for detecting and classifying brain tumors using CNN, LBP, and a multi-layered SVM approach. Priyanka et al. (Modiya and Vahora, 2022) presented an enhanced MR-based brain tumor classification methodology using EfficientNet-B7 with transfer learning and PCA. Srinivas et al. (2022) proposed a pre-trained CNN model such as VGG-16 with transfer learning to detect brain abnormality from MR images. Rahman and Islam (2023) implemented a parallel deep CNN (PDCNN) architecture for detecting MR-based brain tumors.

### 1.1. Research gaps

From an analysis of the above literature, the following observations emerged.

(1) Some methods used wavelet-based feature extraction techniques but showed poor directionality and introduced artifacts around edges. Therefore, they fail to extract complete texture details at the edges.
(2) Some mechanisms utilize second- and higher-order statistical texture features such as GLCM and GLRLM. However, they ignore local texture details because they omit an image’s spatial relationship between distinct local texture patterns.
(3) A few approaches have applied LBP and its variants to extract meaningful local texture information. However, they failed to establish the relationship between central pixels and outside neighborhood pixels. As a result, some significant texture features could be lost.
(4) A few models recommend CNN-based brain tumor detection approaches, but these require a lot of data to train the model, and high computational complexity. Besides that, selecting the number of convolutional layers, epochs, and other training parameters like batch size, optimization technique, and the learning rate is a difficult task.
(5) In most approaches, conventional image enhancement techniques, such as median and average filters, and histogram equalization, were used to remove unwanted image noise. However, some fine details in the image must be recovered in their approaches.

To address the problems reported above, we propose a new approach for detecting and differentiating brain MR images based on tuned single-scale retinex (TSSR), entropy-based thresholding, non-local binary pattern (NLBP) descriptor, and supervised learning approaches such as SVM, KNN, RF and GentleBoost (GB).

### 1.2. Highlights of the proposed study

The significant contributions of the proposed approach are as follows:

(1) A retinex model is utilized to enhance the brightness/contrast of brain MR images to improve the suggested segmentation and classification model’s performance. The significant advantage of this approach is describing human visual perception.
(2) Maximum entropy-based image thresholding and morphological operations are employed to differentiate tumor and non-tumor areas of brain MR images. This process significantly extracts both single and multiple tumor regions.
(3) We implemented a new feature descriptor such as NLBP to extract meaningful texture details for efficient classification. This technique considers the structural relationships between a local patch and a global image. As a result, we can adequately capture hidden information in the image by interacting with pixels outside the neighborhood region, which is a significant contribution to our proposed model.
(4) To accurately estimate the model’s performance, we employed K-fold cross-validation since it minimizes the bias and reduces the computational cost.

The rest of the work is organized as follows: In Section “2. Methodology,” we explore the description of the proposed technique, which includes tumor segmentation, feature extraction, and classification. In “Section 3. Results and discussion,” the segmentation and classification outcomes of both the presented and existing approaches are analyzed, and finally, in Section “4. Conclusion and future scope,” we describe the conclusion of the proposed system.

## 2. Methodology

**FIGURE 1:**
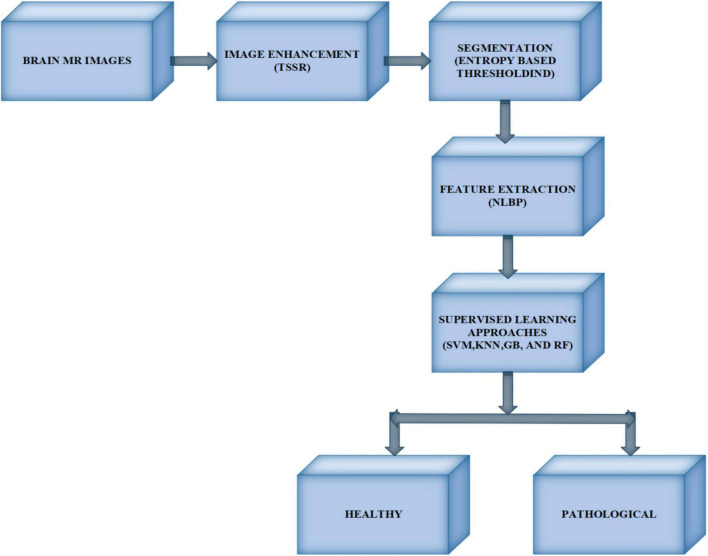
Block diagram of the proposed framework.

The proposed model includes four modules: image enhancement using TSSR, image segmentation by entropy-based thresholding along with morphological operations, feature extraction with the NLBP texture descriptor, and classification using SVM, KNN, RF, and GB, as shown in Figure 1.

### 2.1. Image enhancement

**FIGURE 2:**
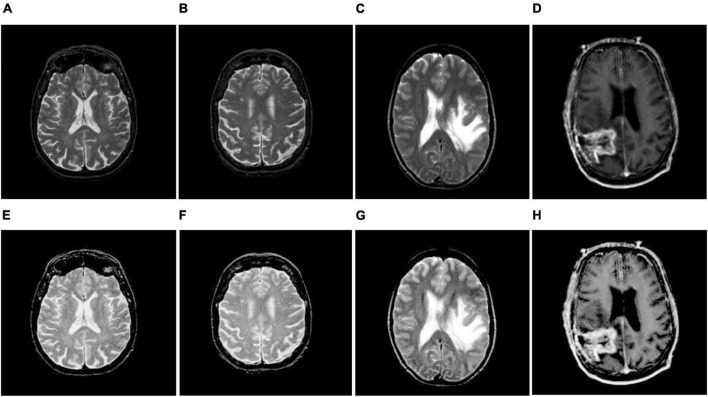
Brain MR image enhancement. **(A–D)** Original brain MR images; **(E–H)** enhanced brain MR images.

**FIGURE 3:**
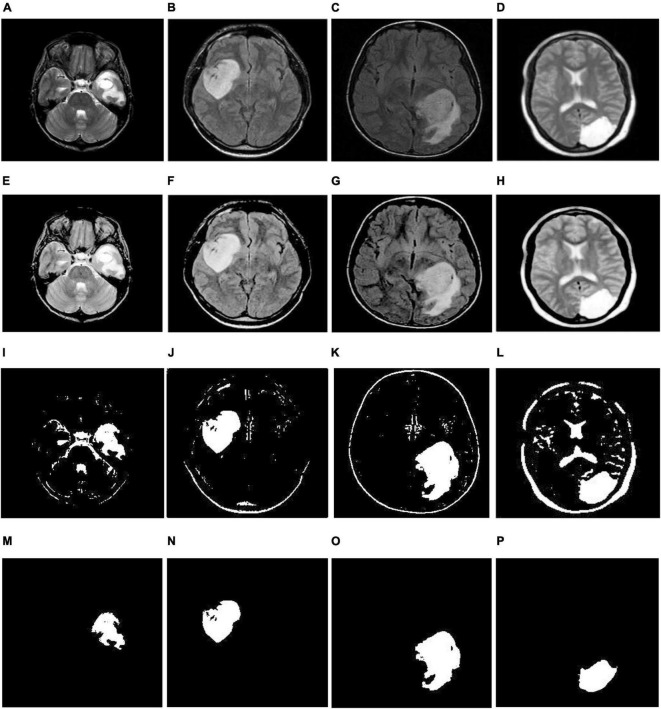
Illustration of the suggested segmentation approach (single tumor): **(A–D)** Input images; **(E,H)** enhancement using TSSR; **(I–L)** lumor extraction by entropy-based thresholding; **(M–P)** post-processing using mathematical morphology.

**FIGURE 4:**
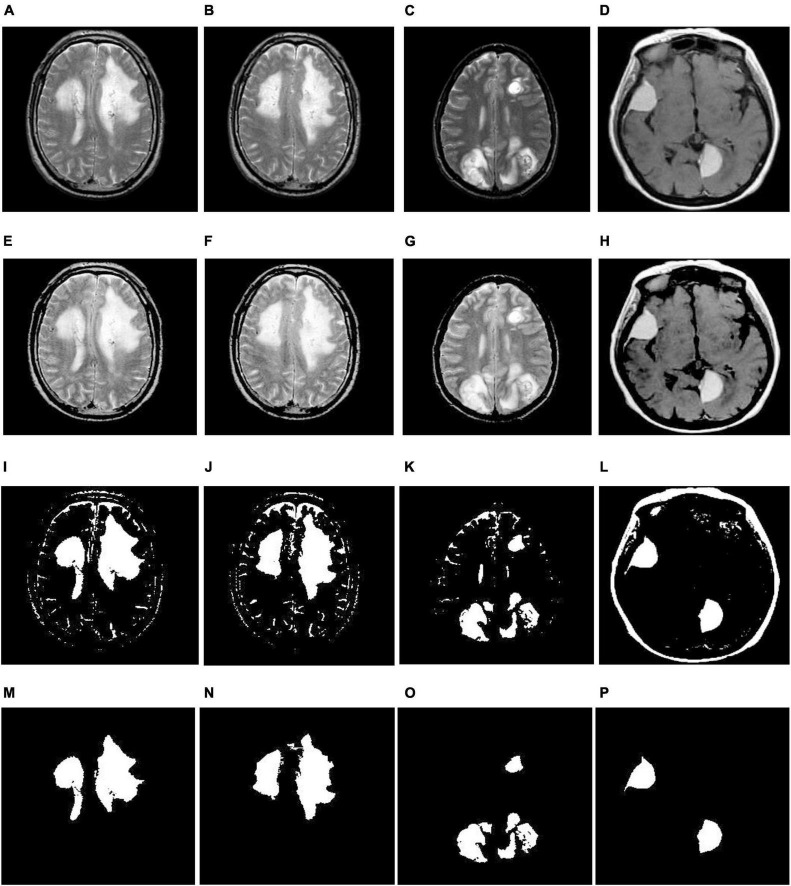
Illustration of the suggested segmentation approach (multiple tumors): **(A–D)** Input images; **(E–H)** enhancement using TSSR; **(I–L)** tumor extraction by entropy-based thresholding; **(M–P)** post-processing using mathematical morphology.

The MR imaging sequence is an essential diagnostic tool for analyzing medical images, and it is used to visualize the inherent structure of the human body. However, during the acquisition process, MR images suffer from several types of image noise (Boyat and Joshi, 2015) due to environmental factors, particularly illumination. Thus, the input images may lose precise information and reduce image quality, severely impacting practitioners during disease examination. Therefore, acquiring good-quality MR images is vital in improving detection accuracy. For this purpose, a tuned single-scale retinex (TSSR) approach has been implemented (Al-Ameen and Sulong, 2016). With this method, the contrast and brightness of MR brain images greatly improved without losing fine details compared to existing systems (Garg and Juneja, 2019). The mathematical representation of the TSSR is as follows:


(1) $$T\,S\,S\,R\,\left(x,y\right)=\log\left(M\,\left(x,y\right)\right)-\log\left(g\,{\left(x,y\right)}^{*}\,M\,\left(x,y\right)\right),$$

(2) $$g\,\left(x,y\right)=\frac{K\,\exp\left(-\frac{W\,{\left(x,y\right)}^{2}+V\,{\left(x,y\right)}^{2}}{L^{2}}\right)}{\xi },$$

(3) $$K=\frac{1}{\sum_{x=1}^{L}\sum_{y=1}^{L}\exp\left(-\frac{W\,{\left(x,y\right)}^{2}+V\,{\left(x,y\right)}^{2}}{L^{2}}\right)},$$

where *M*(*x*,*y*) is the original image; *L* represents the dimensions of the image; *W* and *V* represent the gradient along the *x* and *y* directions, respectively; *K* denotes the normalization factor; *g*(*x*,*y*) signifies the modified Gaussian function; ‘*’ indicates the convolution operator; and ξ is an arbitrary constant that controls the brightness and contrast of an image. In this work, we chose ξ as 1.5. The resulting outcomes of TSSR are shown in Figure 2 as well as Figures 3, 4A–H. We observed that using the TSSR method, we improve the visual details of the affected tumor region compared to source images. As a result, the proposed model effectively detects and distinguishes tumors from enhanced MR brain images. After that, we performed image segmentation to extract the infected area of the brain tumor.

### 2.2. Image segmentation

Image segmentation is a frequently used approach in medical imaging to assist radiologists during the treatment planning of brain tumors. Here, the main objective is to obtain the region-of-interest (ROI) or infected tumor area from MR-based brain tumors. Recently, several segmentation frameworks have been introduced (Rao and Karunakara, 2021). However, it is challenging due to the low sensitivity of boundary pixels and tumor characteristic variations. Therefore, maximum entropy-based image thresholding and morphological operations have been introduced. The following steps are involved in the proposed brain tumor segmentation:

(1) First, we estimate the histogram of the source image *I* and then calculate the probability *p* of each grayscale.
(2) Divide the image *I* into two regions *R*_1_ (foreground) and *R*_2_ (background) over a threshold, *t*(0 < =*t* < *L*−1). Here, *L*is the number of intensity levels.
(3) Evaluate the probability density function of *R*_1_ and *R*_2_ as follows:

(4) $$R_{1}\to \left(\frac{p\,\left(0\right)}{P_{1}\,\left(t\right)},\frac{p\,\left(1\right)}{P_{1}\,\left(t\right)},\frac{p\,\left(2\right)}{P_{1}\,\left(t\right)},\frac{p\,\left(3\right)}{P_{1}\,\left(t\right)},\ldots ,\frac{p\,\left(t\right)}{P_{1}\,\left(t\right)},0,\ldots \,0\right),$$

(5) $$R_{2}\to (0,0\ldots 0,\frac{p\,\left(t+1\right)}{P_{2}\,\left(t\right)},\frac{p\,\left(t+2\right)}{P_{2}\,\left(t\right)},\frac{p\,\left(t+3\right)}{P_{2}\,\left(t\right)},\frac{p\,\left(t+4\right)}{P_{2}\,\left(t\right)},$$

$$\;\ldots ,\frac{p\,\left(L-1\right)}{P_{2}\,\left(t\right)}),$$

where, $P_{1}\,\left(t\right)=\sum_{k=0}^{t}p\,\left(k\right)$ and $P_{2}\,\left(t\right)=\sum_{k=t+1}^{L-1}p\,\left(k\right)$

(4) Under each threshold value *t*, calculate the total entropy *H*

(6) $$H\,\left(t\right)=H_{1}\,\left(t\right)+H_{2}\,\left(t\right),$$

where, *H*_1_, *H*_2_ are the entropies of *R*_1_, *R*_2_, and they are estimated by

(7) $$H_{1}\,\left(t\right)=-\sum_{k=0}^{t}\frac{p\,\left(t\right)}{P_{1}\,\left(t\right)}\,\log\left(\frac{p\,\left(t\right)}{P_{1}\,\left(t\right)}\right)$$

(8) $$H_{2}\,\left(t\right)=-\sum_{k=t+1}^{L-1}\frac{p\,\left(t\right)}{P_{2}\,\left(t\right)}\,\log\left(\frac{p\,\left(t\right)}{P_{2}\,\left(t\right)}\right)$$

(5) Evaluate the optimal threshold value, *T*_*opt*_ by choosing the maximum entropy *H*_*max*_ and then divide it by the maximum pixel intensity value of an image.

(9) $$T_{o\,p\,t}=\frac{H_{\max}}{255}$$

(6) We separate the infected area from MR images based on the thresholding value obtained in step 5, and the corresponding implications are shown in Figures 3, 4I–L. From there, we observe that we relatively extract the ROI (tumor) during this process, but there is a scope for enhancement since they continue to show unnecessary spurious spots or dots.

(7) Finally, we employed mathematical morphological operations (Gonzalez et al., 2003) to remove the imperfections that appeared in step 6. To achieve this, we employed erosion followed by dilation (opening) on a thresholding image with the help of a disk-shaped structuring element over a range of radius 8-12. The corresponding outcomes are illustrated in Figures 3, 4M–P. From this, we note that the proposed segmentation approach separates a single tumor and multiple tumors from the enhanced brain MR images. As a result, we can improve the classification accuracy by extracting the relevant features.

### 2.3. Feature extraction

Texture features play a vital role in classification of medical images since they can significantly capture the variations within the image. The available techniques utilize local-binary patterns (LBP) and their variants extensively (Maheshwari et al., 2019, 2021; Maheshwari and Kumar, 2022). Most approaches extract the inherent texture details by encoding the intensity difference between a central pixel and its neighborhoods. However, they fail to capture long-distance pixel interactions outside the defined neighborhood. Therefore, they need to fully consider the structural relationship between the entire image and its local image patch. It can be overcome by a newly developed texture descriptor known as NLBP (Song et al., 2020) and is mainly used to encode the non-uniform patterns analogous to complex structures within the image, such as corners, lines, and edges.

In the NLBP descriptor process, we initially estimate the non-local central pixels (or anchors) using global image statistics. Then, we encode the intensity difference between anchors and their neighborhoods non-locally using extended rotation-invariant uniform (*eriu*2) patterns. The entire procedure of the NLBP descriptor is outlined as follows:

(1) Primarily, arrange the intensity values of the central pixels of the segmented image *S* in ascending order as follows:

(10) $$\left({\bar{i}}_{c_{1}},{\bar{i}}_{c_{2}},{\bar{i}}_{c_{3}},\ldots ,{\bar{i}}_{c_{n}},\ldots ,{\bar{i}}_{c_{N}}\right)$$

(11) $$:=s\,o\,r\,t\,\left(i_{c_{1}},i_{c_{2}},i_{c_{3}},\ldots ,i_{c_{n}},\ldots ,i_{c_{N}}\right)$$

where, “sort (.)” represents the sorting function, ${\bar{i}}_{c_{n}}$ denotes the intensity of the *n*-th sorted central pixel, and *N*is the number of central pixels.

(2) Partition the sorted central pixels into *M*equal intervals and then compute the intensities of anchors over *M*intervals:

(12) i˜am=Σn=(m−1)⌊NM⌋+1m⌊NM⌋i˜cn⌊NM⌋

where “⌊.⌋ “ denotes the greatest integer function and ${\tilde{i}}_{a_{m}}$ gives the intensity of the *m*−*th* anchor.
(3) Finally, we employed “*eriu*2” to encode the non-local intensity difference between anchors and their neighborhoods as follows:

(13) $$N\,L\,B\,P_{r,K,m}^{e\,r\,i\,u\,2}=\left\{ \begin{array}{c} \sum_{k=0}^{K-1}f\,\left(i_{r,k}-{\tilde{i}}_{a_{m}}\right);U\,\left(N\,L\,B\,P\right)\leq 2\\ K+1;U\,\left(N\,L\,B\,P\right)=4\\ K+2;U\,\left(N\,L\,B\,P\right)=6\\ K+3;U\,\left(N\,L\,B\,P\right)=8\\ K+4;U\,\left(N\,L\,B\,P\right)=10\\ K+5;e\,l\,s\,e\,w\,h\,e\,r\,e \end{array}\right.,$$

where “*r*” is the radius, “*K*” denotes the number of neighboring samples, and
  “*U*” is determined by Eq. (10).

(14) $$U\,\left(N\,L\,B\,P\right)=\left|f\,\left(i_{r,K-1}-{\tilde{i}}_{a_{m}}\right)-f\,\left(i_{r,0}-{\tilde{i}}_{a_{m}}\right)\right|$$

$$+\sum_{k=0}^{K-1}\left|f\,\left(i_{r,k}-{\tilde{i}}_{a_{m}}\right)-f\,\left(i_{r,k-1}-{\tilde{i}}_{a_{m}}\right)\right|$$

**FIGURE 5:**
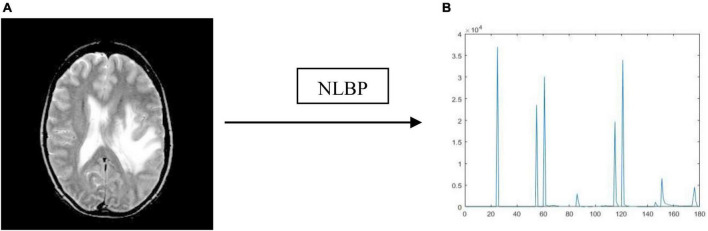
Resultant outcome of NLBP descriptor: **(A)** Source image; **(B)** histogram of NLBP.

Thus, by the NLBP descriptor, we attain *K* + 6 patterns for each central pixel over an anchor. In this work, we consider *r* as five, and *K* as twenty-four for an effective classification. Figure 5 represents the histogram of the NLBP feature descriptor.

### 2.4. Classification

Classification plays a significant role in processing medical images, especially when identifying brain MR image abnormalities. A proper selection of learning algorithms can accomplish this. In this work, we consider support vector machine (SVM), K-nearest neighbors (KNN), random forest (RF), and GentleBoost (GB).

#### 2.4.1. Support vector machine (SVM)

Currently, SVM (Vapnik, 2013) is commonly used in the neuroimaging analysis, particularly in classification problems. The primary objective of this approach is to estimate an optimal hyperplane by maximizing the margin between data points and the decision surface. However, in most cases, the data points within the classes are contaminated by noise, where SVM (linear SVM) cannot distinguish the data points completely. To limit this issue, introducing a *soft margin* and a cost function, *C* into SVM helps to mitigate the training error (Cortes and Vapnik, 1995). In some scenarios, especially when we map a feature set that contains many features into a higher dimensional feature space, the data points within the classes are not linearly separable. For this reason, a nonlinear SVM is initiated with the help of a kernel trick (Cristianini and Shawe-Taylor, 2000). In this paper, we have chosen a radial basis function (RBF) kernel because:

(1) It provides good performance when the feature set contains fewer features.
(2) It needs fewer hyperparameters compared to the polynomial kernel.

The RBF kernel is defined by the following equation


(15) $$F\,\left(y_{i},y_{j}\right)=\exp\left(-\alpha \,{\left|y_{i}-y_{j}\right|}^{2}\right),\alpha >0$$

where, *y*_*i*_ and *y*_*j*_ denote the objects *i* and *j*; α denotes the kernel variable which is used to evaluate the smoothness of the boundary between the classes in the original object space.

#### 2.4.2. GentleBoost (GB)

GentleBoost (GB) (Schapire and Singer, 1999) is formally known as Gentle adaptive boosting or Gentle AdaBoost and is developed by integrating the features of AdaBoost and LogitBoost. Let us assume that, *X* = [(*x*_1_,*y*_1_),(*x*_2_,*y*_2_),…,(*x*_*P*_,*y*_*P*_)] is a training vector with *P* number of attributes and the corresponding label associated with input features *x*_*q*_ is *y*_*m*_ = [−1,1]. Here, we perform binomial classification, using the decision tree as a weak classifier. The procedure for obtaining a strong classifier with GentleBoost is outlined in Algorithm 1.

**Algorithm 1:** GentleBoost

1. Begin: Initialize the weights as $g_{q,1}=\frac{1}{P}$ ; *q* = 1,2,3,…,*P* 2. Repeat the following steps for each round *v* = 1,2,3,…,*V* (b) To train the decision tree (weak classifier), subdivide the training set *X* into *D* number of partitions. Then, for each $X_{v}^{j}$ partition (here *j* = 1,2,3,…,*D*), calculate $g_{v+}^{j}$ and $g_{v-}^{j}$ using (16) $$g_{v+}^{j}=\sum_{q:x_{q}\in X_{v}^{i}\,\wedge \,y_{q}=1}g_{q,v}$$ (17) $$g_{v-}^{j}=\sum_{q:x_{q}\in X_{v}^{i}\,\wedge \,y_{q}=-1}g_{q,v}$$ (c) Estimate the weak hypothesis for each $X_{v}^{j}$ as follows (18) $$h_{v}^{b}\,\left(x\right)=\frac{\left(g_{v+}^{j}-g_{v-}^{j}\right)}{g_{v+}^{j}+g_{v-}^{j}}$$ (d) Update the weights using Eq. (15) and Eq. (16) (19) $$g_{q,v+1}=\frac{g_{q,v}\,\exp\left[y_{q}\,h_{v}\,\left(x_{q}\right)\right]}{W_{v}},$$ (20) $$W_{v}=\sum_{q}g_{q,v}\,\exp\left[y_{q}\,h_{v}\,\left(x_{q}\right)\right]$$ 3. Obtain the optimal hypothesis to classify brain MR images as healthy or pathological by (21) $$H\,\left(x_{q}\right)=\text{sign}\,\left[\sum_{v=1}^{V}h_{v}\,\left(x_{q}\right)\right]$$

The hyperparameters of the GB classifier as follows:

(1) The maximum number of splits allowed by classification tree = 10.
(2) Learner = ‘Decision tree’.
(3) Number of decision trees = 100

#### 2.4.3. K- nearest neighbor (KNN)

KNN (Cunningham and Delany, 2007) is a widely supervised machine learning approach for analyzing both classification and regression problems. Moreover, it is primarily applied in classification scenarios. Typically, KNN stores all data points of corresponding classes and then distinguishes the new classes based on the distance metrics between the data points.

Generally, in KNN, the classes are separated by a majority vote of their neighbors. Based on this majority vote, KNN predicts the most frequent classes among those with the help of distance metrics. In KNN, the selection of *K* and the distance function plays a prominent role in improving classification accuracy.

(1) The first criterion is a selection of *K*; if the dataset is small, then choose a smaller *K*value; otherwise choose a higher value of *K*. In this work, we chose_*K*_ as 3.
(2) A second criterion is a distance function. Several distance functions estimate the similarity between data points, such as Euclidean, Manhattan, Mahalanobis, and Chebyshev. In this work, we preferred Euclidean distance because it provides reasonable accuracy for categorizing categorical data.

#### 2.4.4. Random forest (RF)

The RF (Liaw and Wiener, 2002) is used to tackle classification and regression problems. It employs ensemble learning, combining multiple classifiers to solve complex problems. An RF comprises several decision trees and creates a “forest” trained by bagging or bootstrap aggregation. Bagging is a meta-algorithm for ensembles that increases the efficiency of machine learning methods. The outcome in RF is determined based on the predictions of the decision trees. It makes predictions by taking the average, or “mean,” of the results from several trees. The model’s efficiency grows as the number of trees increases but sometimes may lead to overfitting. Finally, the constraints of decision trees are eliminated by RF. This further decrease the dataset’s overfitting and boosts accuracy.

The characteristics of the RF learning approach are as follows:

(1) Learner = ‘Decision tree’.
(2) Number of estimators = 100.

### 2.5. Evaluation measures

The performance of the proposed framework is validated using various well-known metrics, such as the true positive rate (TPR), true negative rate (TNR), positive predictive value (PPV), F-score, dice similarity coefficient (DSC), area under the curve (AUC), and accuracy (Raschka, 2018).

## 3. Results and discussion

### 3.1. Database

To validate the performance of the suggested and existing frameworks, 235 two-dimensional T2-weighted MR-based brain images were taken, including 45 healthy and 190 pathological images with dimensions of 256 × 256 from an open access benchmark database such as Harvard Medical School (Johnson and Becker, n.d.).

### 3.2. Simulation results

This result division is subdivided into two modules to emphasize the detection (or segmentation) and classification independently. Here, the first module identifies tumor areas by the image segmentation process, and the second module engages with the discrimination between healthy and pathological brain MR images using appropriate feature extraction and supervised machine learning approaches.

#### 3.2.1. Identification of the tumor area

In this section, we detect the tumor portions from brain MR images using maximum entropy-based thresholding and morphological operations. Before that, we improved the dynamic range of brain MR images by adopting the human visual perception concept. Due to this, we efficiently preserve fine details by enhancing contrast as well as brightness. As a result, we increase the segmentation accuracy. After that, we separate foreground (tumor area) and background (non-tumor area) regions by maximum entropy-based thresholding. However, through this process, we cannot achieve significant performance since images generated by the thresholding approach are distorted by noise and texture (see Figures 3, 4I–L). Therefore, we employed morphological operations to remove the tiny objects that appeared in the thresholding image without minimizing the size and shape of the large objects (see Figures 3, 4M–P). Through this process, we effectively improve the segmentation performance with 91.88% DSC, 99.71% PPV, 99.38% TPR, 95.69% TNR, 99.55% F-Score, 97.54% AUC and 99.17% accuracy (see Table 1).

**TABLE 1:** Segmentation performance of the proposed approach.

Sample image	DSC	PPV	TPR	TNR	F-Score	AUC	Accuracy
1	97.85	99.87	99.96	96.70	99.91	98.33	99.84
2	89.01	99.85	99.7	92.42	99.77	96.06	99.56
3	83.69	99.76	98.56	94.74	99.15	96.65	98.39
4	92.66	99.47	99.32	93.55	99.39	96.44	98.88
5	85.03	98.28	98.29	82.85	98.53	90.82	97.33
6	87.75	99.52	99.68	85.68	99.6	92.68	99.22
7	90.69	98.96	99.74	85.84	99.35	92.79	98.79
8	95.89	99.65	99.97	92.60	99.81	96.29	99.64
9	91.81	100	99.64	99.92	99.82	99.78	99.65
10	93.75	100	99.47	100	99.73	99.74	99.49
11	94.02	99.96	99.65	98.61	99.8	99.13	99.61
12	95.71	99.32	99.21	95.52	99.26	97.36	98.72
13	91.81	99.99	98.12	99.87	99.04	98.99	98.28
14	88.88	99.62	98.8	94.36	99.21	96.58	98.52
15	93.24	99.98	99.2	99.69	99.58	99.44	99.23
16	92.27	99.76	98.96	96.94	99.36	97.95	98.82
17	90.28	100	99.86	99.55	99.92	99.7	99.86
18	91.41	98.86	99.6	87.74	99.23	93.67	98.59
19	94.94	99.99	99.79	99.33	99.88	99.56	99.78
20	87.66	99.99	99.30	99.51	99.64	99.4	99.3
21	94.02	100	99.35	99.91	99.67	99.63	99.38
22	87.04	100	99.1	100	99.55	99.35	99.13
23	90.42	100	99.42	100	99.71	99.71	99.44
24	97.84	99.92	99.97	96.97	99.94	98.47	99.89
25	99.27	100	99.92	100	99.96	99.96	99.92
**Average**	91.88	99.71	99.38	95.69	99.55	97.54	99.17

#### 3.2.2. Classification of brain MR images

**FIGURE 6:**
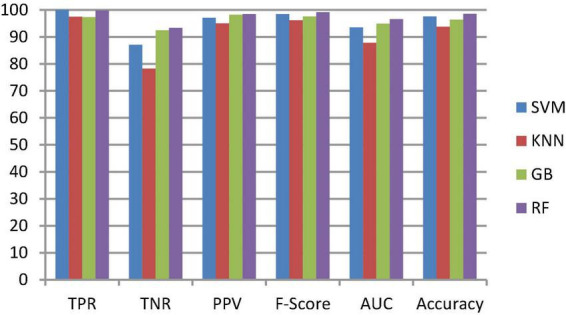
Classification performance comparison between various classifiers without enhancement and segmentation.

**FIGURE 7:**
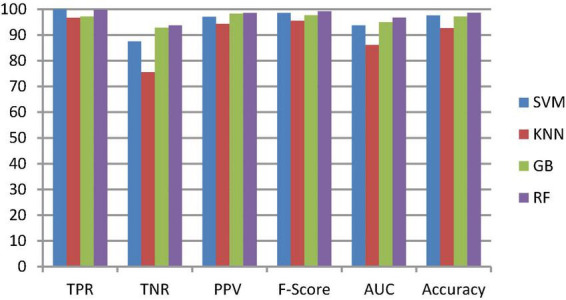
Classification performance comparison between various classifiers with enhancement and without segmentation.

**FIGURE 8:**
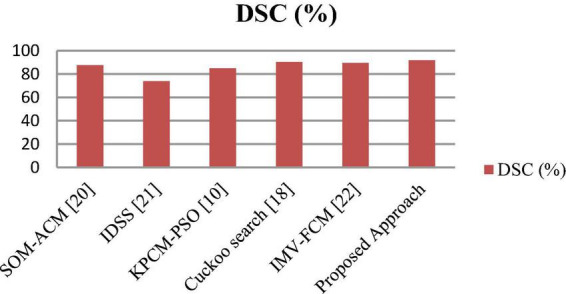
Comparative analysis of the proposed and existing segmentation approaches based on the DSC.

To classify the given brain MR images as healthy or pathological, we extracted relevant features from segmented brain MR images using the NLBP feature descriptor by establishing interactions between anchors and their neighboring pixels. Then, the obtained features were fed to various supervised learning approaches such as SVM, KNN, RF, and GB, and they were assessed through *K*-fold cross-validation (*K*−FCV). Typically, K-FCV is a simple and effective method compared to other validation strategies (Raschka, 2018) that involve a typical resampling technique without any replacement in the data. Furthermore, in each fold of the *K*−FCV train and test would be conducted precisely once during this whole procedure, which helps us avoid overfitting. However, the selection of the *K*-value is a significant factor during validation. A lower number of *K*-folds will produce a model that fits the data poorly and has a strong bias and low variance. Similarly, a high *K*-fold value results in an overfitting model. Therefore, to prevent this uncertainty, we picked a reasonable number of 5 for *K*, and the outcomes are tabulated in Tables 2–4. The corresponding average performance is summarized in Tables 5–7, as well as in Figures 6–8. From there, we observed that in cases involving enhancement and segmentation, the proposed approach achieved high classification accuracy when we employed the RF classifier (see Table 7) compared to other cases (see Tables 5, 6) since by segmenting the brain MR images, we efficiently distinguished the characteristics of pathological and healthy tissues.

**TABLE 2:** Classification performance of the implemented framework without enhancement and segmentation using 5-FCV.

Classifier	5-FCV	TPR	TNR	PPV	F-Score	AUC	Accuracy
SVM	1st Fold	100	9111	97.94	98.96	95.55	98.29
	2nd Fold	100	86.67	96.94	98.45	93.33	97.45
	3rd Fold	100	84.44	96.45	98.19	92.22	97.02
	4th Fold	100	86.67	96.94	98.45	93.33	97.45
	5th Fold	100	86.67	96.94	98.45	93.33	97.45
KNN	1st Fold	96.84	77.78	94.84	95.83	87.31	93.19
	2nd Fold	97.37	82.22	95.85	96.6	89.79	94.47
	3rd Fold	98.42	75.55	94.44	96.38	86.98	94.04
	4th Fold	97.37	80	95.36	96.35	88.68	94.04
	5th Fold	97.37	75.55	94.38	95.85	86.46	93.19
GB	1st Fold	93.68	93.33	98.34	94.97	93.5	93.61
	2nd Fold	98.95	91.11	97.92	98.43	95.03	97.44
	3rd Fold	97.89	88.89	97.38	97.63	93.39	96.17
	4th Fold	97.89	97.78	99.46	98.67	97.83	97.87
	5th Fold	98.42	91.11	97.9	98.16	94.76	97.02
RF	1st Fold	100	95.55	98.96	99.47	97.77	99.15
	2nd Fold	99.47	95.55	98.95	99.21	97.51	98.72
	3rd Fold	100	86.67	96.64	98.44	93.33	97.44
	4th Fold	99.47	91.11	97.93	98.69	95.29	97.87
	5th Fold	100	97.78	9947	99.73	98.89	99.57

**TABLE 3:** Classification performance of the presented approach with enhancement and without segmentation using 5-FCV.

Classifier	5-FCV	TPR	TNR	PPV	*F*-Score	AUC	Accuracy
SVM	1st Fold	100	88.89	97.43	98.69	94.44	97.87
	2nd Fold	100	91.11	97.94	98.96	95.5	98.29
	3rd Fold	100	88.89	97.43	98.69	94.44	97.87
	4th Fold	100	84.44	96.44	98.18	92.22	97.02
	5th Fold	100	84.44	96.44	98.18	92.22	97.02
KNN	1st Fold	97.37	75.55	94.38	95.85	86.46	93.19
	2nd Fold	96.84	75.55	94.36	95.58	86.19	92.76
	3rd Fold	96.31	73.33	93.84	95.06	84.82	91.91
	4th Fold	97.89	75.55	94.41	96.12	86.72	93.62
	5th Fold	95.26	77.77	94.7	95.01	86.51	91.91
GB	1st Fold	97.89	88.89	97.38	97.63	93.39	96.17
	2nd Fold	96.84	95.55	98.95	97.88	96.19	98.72
	3rd Fold	96.31	88.89	97.41	96.85	92.6	97.02
	4th Fold	98.42	93.33	98.42	98.42	95.87	97.44
	5th Fold	96.31	97.78	99.45	97.85	97.64	96.59
RF	1st Fold	100	95.55	98.96	99.47	97.77	99.15
	2nd Fold	100	95.55	98.96	99.47	97.77	99.15
	3rd Fold	100	93.33	98.44	99.21	96.66	98.72
	4th Fold	100	91.11	97.94	98.96	95.55	98.29
	5th Fold	98.95	93.33	98.44	98.95	96.14	97.87

**TABLE 4:** Classification performance of the proposed approach with enhancement and segmentation using 5-FCV.

Classifier	5-FCV	TPR	TNR	PPV	F-Score	AUC	Accuracy
SVM	1st Fold	100	91.11	97.94	98.96	95.55	98.29
	2nd Fold	99.47	86.67	96.92	98.33	93.07	97.02
	3rd Fold	100	88.86	97.43	98.69	94.44	97.87
	4th Fold	100	91.11	97.94	98.96	95.55	98.29
	5th Fold	100	91.11	97.94	98.96	95.55	98.29
KNN	1st Fold	100	93.33	98.44	99.21	99.66	98.72
	2nd Fold	100	88.89	97.43	98.69	94.44	97.87
	3rd Fold	99.47	100	100	99.73	99.73	99.57
	4th Fold	100	91.11	97.94	98.96	95.55	98.29
	5th Fold	99.47	93.33	98.43	98.95	96.4	98.29
GB	1st Fold	100	95.55	98.96	99.47	97.77	99.15
	2nd Fold	99.47	95.55	98.95	99.21	97.51	98.72
	3rd Fold	99.47	100	100	99.73	99.73	99.57
	4th Fold	98.95	97.78	9.47	99.21	98.36	98.72
	5th Fold	98.95	97.78	9.47	99.21	98.36	98.72
RF	1st Fold	100	97.78	99.47	99.73	98.89	99.57
	2nd Fold	100	97.78	99.47	99.73	98.89	99.57
	3rd Fold	99.47	100	100	99.73	99.73	99.57
	4th Fold	100	100	100	100	100	100
	5th Fold	100	100	100	100	100	100

**TABLE 5:** Average classification performance of the proposed framework without enhancement and segmentation.

Classifier	Evaluation measures (%)					
	**TPR**	**TNR**	**PPV**	**F-Score**	**AUC**	**Accuracy**
SVM	100	87.11	97.04	98.5	93.55	97.53
KNN	97.47	78.22	94.97	96.2	87.84	93.78
GB	97.36	92.44	98.2	97.57	94.9	96.42
RF	99.78	93.33	98.45	99.11	96.56	98.55

**TABLE 6:** Average classification performance of the proposed framework with enhancement and without segmentation.

Classifier	Evaluation measures (%)					
	**TPR**	**TNR**	**PPV**	***F*** **-Score**	**AUC**	**Accuracy**
SVM	100	87.55	97.14	98.54	93.77	97.61
KNN	96.73	75.55	94.35	95.52	86.14	92.68
GB	97.15	92.88	98.32	97.72	95.02	97.18
RF	99.79	93.77	98.55	99.21	96.78	98.64

**TABLE 7:** Average classification performance of the suggested method with enhancement and segmentation.

Classifier	Evaluation measures (%)					
	**TPR**	**TNR**	**PPV**	**F-Score**	**AUC**	**Accuracy**
SVM	99.89	89.78	97.63	98.78	94.83	97.95
KNN	99.78	93.33	98.45	99.11	97.15	98.55
GB	99.37	97.33	99.37	99.36	98.35	98.98
RF	99.89	99.11	99.78	99.84	99.5	99.75

#### 3.2.3. Ablation study

This section talks about the results of ablation studies we did to choose the suggested. These results are illustrated in Tables 5–7. It’s important to note that these results are attained by taking features from enhanced or non-enhanced images and with/without segmentation. The first ablation study (Table 6) described how various ML models are accurate when images are not enhanced and segmented. The results of this study are used as a baseline for the second ablation study. Here, features are directly fed to classifiers without any enhancement and segmentation. Due to that, the presented model doesn’t differentiate normal images from abnormal brain MR images in an effective manner, which results in low-classification accuracy.

The second ablation study (Table 3) mainly deals with the effectiveness of the proposed model when images are enhanced but not segmented. In that case, by enhancing the input brain MR images, we can relatively improve the accuracy of the model compared to the results of the first ablation study since retinex theory considers human visual perception. The outcomes of this study can be considered as a paradigm for the third ablation study.

The third ablation study (Table 7) primarily engages the suggested framework’s significance when enhanced and segmented images. In this scenario, the proposed NLBP descriptor relatively extracts the inherent details from segmented images, resulting in supervised learning approaches efficiently distinguishing normal and abnormal brain MR images compared to the results of the first and second ablation studies.

From the above three ablation studies, we conclude that enhancing and segmenting brain MR tumors can easily differentiate normal and abnormal regions from a given image. So, in the third ablation study, we achieved high accuracy compared to other studies.

#### 3.2.4. Comparison with state-of-the-art approaches

**FIGURE 9:**
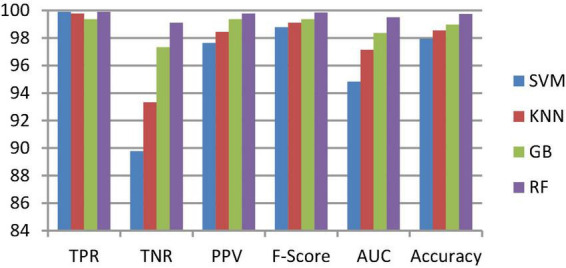
Classification performance comparison between various classifiers with enhancement and segmentation.

**FIGURE 10:**
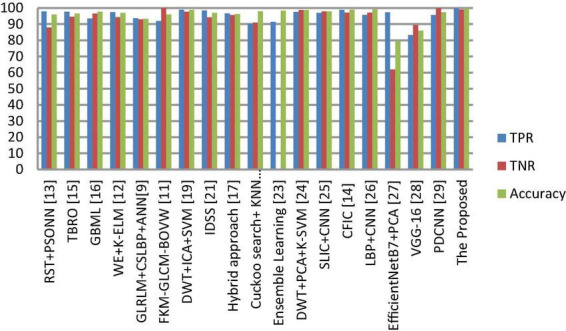
Comprehensive analysis of the proposed and existing classification approaches based on TPR, TNR and accuracy.

The segmentation and classification performance of the proposed system is compared with well-known received approaches, and their results are depicted in Tables 8, 9 as well as Figures 9, 10. From this, it is clear that the suggested framework increases the segmentation and classification accuracy in terms of DSC, TPR, TNR, and accuracy metrics compared to existing works. Hence, the proposed strategy can be used as a diagnostic tool in clinical analysis to help radiologists identify abnormalities in brain MR images. From the analysis of experimental outcomes, we observed that the following parameters play a crucial role in the success of our proposed methodology:

**TABLE 8:** Performance of the presented and existing segmentation frameworks.

Year	Technique	DSC (%)
2020	SOM-ACM (Sandhya et al., 2020)	87.7
2020	IDSS (Polepaka et al., 2020)	74
2021	KPCM-PSO (Sumathi and Mandadi, 2021)	85.05
2021	Cuckoo search (Sumathi et al., 2021)	90.36
2021	IMV-FCM (Hua et al., 2021)	89.61
The proposed approach		91.88

**TABLE 9:** Performance of the presented and existing classification frameworks.

Year	Technique	Metrics (%)		
		**TPR**	**TNR**	**Accuracy**
2018	RST+PSONN (Rajesh et al., 2019)	98	88	96
2018	TBRO (Kanmani and Marikkannu, 2018)	97.76	94.6	96.57
2018	GBML (Selvapandian and Manivannan, 2018)	93.46	96.54	97.75
2018	WE+K-ELM (Lu et al., 2018)	97.48	94.44	97.04
2020	GLRLM+CSLBP+ANN (Mudda et al., 2020)	93.75	93.02	93.4
2020	FKM-GLCM-BOVW (Paul and Sivarani, 2020)	92	100	96
2020	DWT+ICA+SVM (Singh et al., 2020)	98.97	97.68	98.87
2020	IDSS (Polepaka et al., 2020)	98.48	94.28	97.02
2021	Hybrid approach (Habib et al., 2021)	96.7	95.7	96.3
2021	Cuckoo search+ KNN (Sumathi et al., 2021)	90.3	91	98.12
2022	Ensemble Learning (Younis et al., 2022)	91.4	–	98.41
2022	DWT+PCA+K-SVM (Mandle et al., 2022)	97.65	98.78	98.75
2022	SLIC+CNN (Behera et al., 2022)	97	98	98
2022	CFIC (Veeramuthu et al., 2022)	98.86	97.14	98.97
2022	LBP+CNN (Kolla et al., 2022)	95.73	97.12	99.23
2022	EfficientNetB7+PCA (Modiya and Vahora, 2022)	97.33	62	79.67
2022	VGG-16 (Srinivas et al., 2022)	83.33	89.47	86.04
2023	PDCNN (Rahman and Islam, 2023)	95.65	100	97.33
The proposed approach		99.89	99.11	99.75

(1) Using the TSSR technique, we significantly maintain the fine details of pathological brain MR images by enhancing the brightness and contrast. As a result, we effectively distinguish pathological brain MR images from healthy images (see Table 7).
(2) Using the proposed thresholding process, we relatively differentiate the tumor (either single or multiple) and non-tumor regions with high DSC values compared to existing models (see Table 8) since our method considers the local details and eliminates noise spots in the output image. Therefore, we effectively improve the classification accuracy (see Table 7) compared to other scenarios represented in Tables 5, 6.
(3) Using the NLBP feature extraction technique, we efficiently extracted meaningful details by maintaining the structural relationships between the local image patch and the entire image. Consequently, we obtained better classification performance when we employed the method on segmented images (see Table 7) rather than on original and enhanced brain MR images (see Tables 5, 6).
(4) The suggested NLBP descriptor is some extent robust to illumination changes, and Gaussian noise. Hence, the presented model achieved better results than the existing approaches (see Table 9).

## 4. Conclusion and future scope

The brain is the most complicated structure and is responsible for controlling the human body. Therefore, any disorder within the brain may adversely impact human life. Brain tumors are the acute disorders that arise from the abnormal development of cells in the brain. Among all brain tumors, malignant or cancerous tumors are very harmful to human beings; however, if diagnosed at early stages, the majority of victims may recover. For this purpose, in this article, a new methodology is preferred. The method first employed image enhancement using TSSR and then maximum entropy-based thresholding along with morphological operations were applied to extract the tumor affected region. Finally, feature extraction followed by classification is performed based on NLBP and supervised learning approaches are used to distinguish the given brain MR image as healthy or pathological. The abovementioned process is investigated in the MATLAB R2020a environment using images taken from the Harvard Medical School database. After implementing the system, various performance measures have been utilized to evaluate and compare the efficiency of the suggested method with some other existing systems. From this analysis, we observed that the proposed framework reaches better segmentation and classification accuracy when compared to state-of-the-art approaches. Hence, we conclude that our model can be used as a supportive tool for radiologists during the detection of brain tumors. In the future, our work will extend to the prediction of other medical-related diseases such as Parkinson’s disease, breast, and skin cancer.

## Data availability statement

The original contributions presented in this study are included in the article/supplementary material, further inquiries can be directed to the corresponding author.

## Author contributions

KR: conceptualization, methodology, software, validation, investigation, data creation, and writing—original draft preparation. RB: formal analysis. SP: resources and visualization. KR, DB, and RB: writing—review and editing. DB: supervision and funding acquisition. DB and SP: project administration. All authors read and agreed to the published version of the manuscript.
